# Thermotropic liquid crystalline copolyester fibers according to various heat treatment conditions

**DOI:** 10.1038/s41598-021-91212-4

**Published:** 2021-06-02

**Authors:** Won Jun Lee, Lee Ku Kwac, Hong Gun Kim, Jin-Hae Chang

**Affiliations:** 1 Department of Polymer Science and Engineering, Kumoh National Institute of Technology, Gumi, 39177 Korea; 2 Graduate School of Carbon Convergence Engineering, Jeonju University, Jeonju, 55069 Korea; 3 Institute of Carbon Technology, Jeonju University, Jeonju, 55069 Korea

**Keywords:** Chemistry, Materials science

## Abstract

Thermotropic liquid crystal copolyester (TLCP) was synthesized using a melt polymerization method, with a molar ratio composition of 2,5-diethoxy terephthalic acid (ETA), hydroquinone (HQ), and *p*-hydroxybenzoic acid (HBA) of 1:1:3. TLCP exhibited nematic liquid crystalline mesophase and maintained nematic textures under all heat treatment conditions applied. The synthesized TLCP was processed into fibers using a capillary rheometer. The liquid crystalline mesophase, thermo-mechanical properties, and morphology of TLCP fibers obtained under various heat treatment conditions were investigated. The thermo-mechanical properties of the heat-treated fibers were improved compared to those of the as-spun fibers. The best results were obtained for TLCP fibers annealed at 230 °C for 9 h. The heat-treated fibers showed a well-developed microfiber morphology compared to the as-spun fibers. In the spun fibers, a skin–core morphology was observed regardless of the heat treatment conditions, and a well-developed fiber morphology better than the core area was observed in the skin area. The diameter of the fiber heat-treated at 230 °C for 9 h was approximately 60–110 nm.

## Introduction

Thermotropic liquid crystalline polymers (TLCPs) exhibit excellent thermal properties, low viscosity, and can be processed above their melting point^1, 2^, being widely used as commercial engineering polymers. Several researchers have provided possible explanations for the relationship between the chemical structure of TLCPs and their properties and have compared them with other engineering polymers. In particular, compared to lyotropic polyamides, thermotropic polyesters are attracting more attention because they provide more advantages in the field of synthesis or processing^3^.

Most of the TLCPs and their composites exhibit improved physical properties by including in their main chain rigid rod-shaped monomers, such as *p*-hydroxy benzoic acid (HBA), naphthalene diols or their derivatives, naphthalene dicarboxylic acid isomers, 4,4′-biphenol (BP), hydroquinone (HQ), and terephthalic acid (TPA). TLCPs synthesized using monomers whose basic structures are completely aromatic or linear-shaped benzene rings, such as TPA, HQ, and HBA, have very high melting points and are unprocessable^4–6^. In addition, they are not dissolved in general-purpose solvents^7^.

Furthermore, many researchers have studied methods for lowering the melting point for easy processing and increased solubility^8–11^. For these studies, a method of attaching a bulky substituent to the main chain, introducing a flexible alkyl group, and introducing a monomer with a bent structure and asymmetric monomers was used, and the side chain was also substituted. However, when using these methods, the unique physical properties of the original TLCP are not observed due to deterioration of thermo-mechanical properties and reduced processability. Ballauff^12^ indicated transition temperatures of TLCPs that varied with the length of the dialkoxy side groups in the main chain. It was observed that in one of the monomers, 1,4-phenylene 2,5-dialkoxyterephthalate, the transition temperature gradually decreased as the length of the dialkoxy group increased. In addition, Lenz et al.^13^ and Jin et al.^14^ also reported the change in transition temperature according to the length of various alkyl side chains.

Another method to lower the melting point is to synthesize copolymers using monomers with different structures such as monomers containing flexible side groups and naphthalene diol isomers^15, 16^. Synthesis of a copolymer by adding a well-designed third monomer to a monomer frequently used in a homopolymer can control the transition temperature to broaden the application range of TLCPs. For example, if a monomer containing a side group or meta-substituted monomer or an alkyl group is used, a significantly increased processability can be obtained^17^. However, even in this case, the selection of the monomer structure for the copolymer should be careful not to impair the thermo-mechanical properties and liquid crystalline behavior. In the case of copolymerization using two or more mesogenic monomers, the melting point can be considerably reduced. For example, HBA or 2-hydroxy-6-naphthoic acid produces random copolymeric structures with reduced melting points. The reduction of the melting point by copolymerization with a similar structure was reported by Jackson^16^. According to this study, for poly(*p*-phenylene naphthalene-2,6-dicarboxylate), the melting point decreases from 580 °C to approximately 325 °C when copolymerized with 70% HBA.

Heat treatment processes have long been used to increase the polymer properties. For instance, cotton or acrylic fibers are converted into carbon fibers with excellent thermo-mechanical properties by heat treatment^18^. The heat treatment performed below the melting point of the polymer affects the structure of the molecule to increase crystallinity, and it is mainly used for condensation or step growth polymers such as polyesters and polyamides. After heat treatment, the molecular weights of various polyesters and nylons generally increase more than those synthesized using conventional solution or melt polymerization techniques. This technique is called solid-state polymerization, and it is mainly used to increase the molecular weight of nylon or poly(ethylene terephthalate) chips to expand their applications^19–21^.

Drawing is used to improve the final properties of many as-spun fibers. However, the thermo-mechanical properties of TLCP fibers with a straight and rigid-rod type molecular structure are not significantly improved because they slightly change the shape of the molecule through the drawing process. In contrast, the physical properties of the TLCP fiber can be considerably improved through heat treatment processing. The annealing process provides high tensile strength, modulus of elasticity, degree of crystallinity, and molecular weight increase in TLCP fibers^22^. However, it also presents problems in solubility and spinning.

We have already synthesized liquid crystalline (LC) copolyesters using monomers of 2,5-diethoxy terephthalic acid (ETA), HQ, and HBA, increasing the molar ratio of HBA from 0 to 5^11^. These TLCPs showed different thermal and LC properties according to the number of moles of HBA. When the number of moles was 3 mol, the copolyester exhibited the lowest melting point and excellent LC mesophase. From these results, TLCP containing 3 mol of HBA was expected to be the most easily spun, and the physical properties of the obtained fiber were expected to be the best.

In this study, TLCP was synthesized by melt polymerization using a molar ratio of ETA:HQ:HBA = 1:1:3. The fibers were obtained through melt spinning, and the obtained fibers were heat treated at various temperatures and times.

Herein, we will discuss the thermo-mechanical properties, LC mesophase, and morphologies of the fibers finally obtained through the flow behavior of TLCP under specific spinning conditions. In addition, we will show TLCP fibers with optimal properties that appear according to various heat treatment times and temperature conditions. Also, the physical properties of the fibers obtained according to various heat treatment conditions were compared with each other.

## Experimental

### Materials

**Figure 1:**
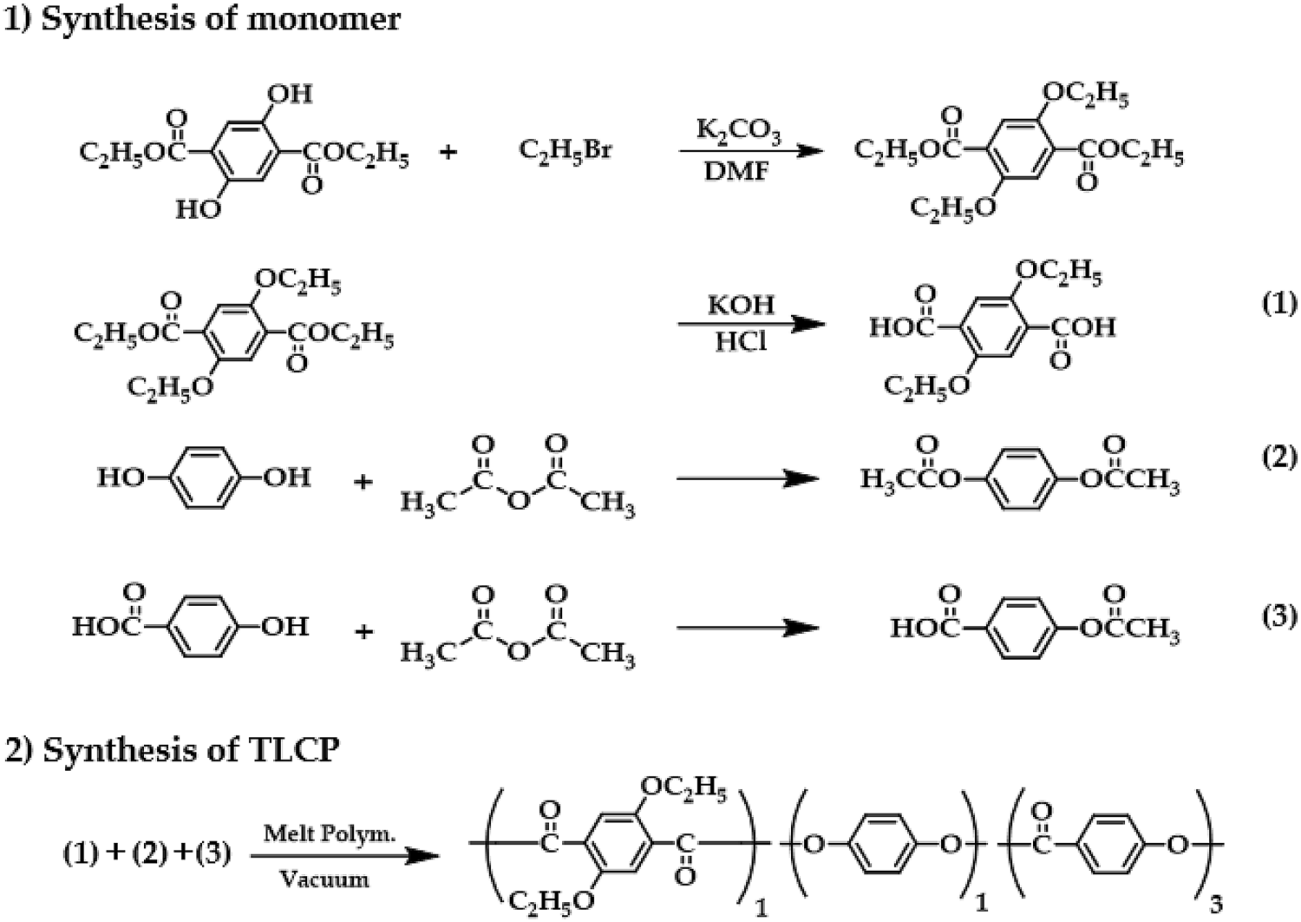
Synthetic routes of TLCPs.

All reagents were purchased from Aldrich Chemical Co. (Yongin, Korea) and TCI (Seoul, Korea) and used without purification; however, a general-purpose solvent was used after purification. TLCPs were synthesized according to the conditions of previous studies^11^. The detailed synthesis method is shown in Fig. 1. To increase the molecular weight for spinning, this sample was synthesized under different conditions than the previously polymerization method. The detailed conditions are summarized in Table 1.

**Table 1:** Melt polymerization conditions of TLCPs.

TLCP	Temperature (°C)/time (min)/pressure (Torr)
	240/120/760 → 260/120/760 → 280/90/760 → 300/65/760 → 310/45/300 → 310/40/1

The synthesized TLCPs were insoluble in almost all common solvents. In particular, they were not dissolved in a mixed solvent such as phenol/*p*-chlorophenol/1,1,2,2-tetrachloroethane = 25:40:35 (w/w/w), which has been mainly used for TLCP dissolution, as shown in Table 2. For convenience, TLCP samples were marked as 190/3 and 230/9. Here, 190 and 230 represent the heat treatment temperatures (°C), and 3 and 9 represent the annealing times (h).

**Table 2:** General properties of heat-treated TLCP fibers.

Heat treat. (°C/h)	IV^a^	T_g_ (°C)	T_m_ (°C)	T_i_ (°C)	ΔT_i−m_^b^ (°C)	T_D_^ic^ (°C)	wt_R_^600d^ (%)	LC phase	DC^e^ (%)
As-spun	Insol.^f^	82	277	315	38	363	35	N^g^	27
190/3	Insol	92	279	317	38	366	34	N	30
210/3	Insol	94	280	317	37	367	35	N	36
230/3	Insol	98	280	322	42	366	35	N	36
250/3	Insol	97	287	321	34	367	36	N	37
230/3	Insol	98	280	322	42	366	35	N	36
230/6	Insol	98	281	322	41	366	35	N	37
230/9	Insol	102	286	325	41	363	35	N	36
230/12	Insol	93	276	318	42	363	36	N	33

^a^Inherent viscosity was measured at a concentration of 0.1 g/dL solution in phenol/*p*-chlorophenol/TCE = 25/40/35 (w/w/w) at 25 °C.

^b^Temperature ranges of the liquid crystalline mesophase.

^c^At a 2% initial weight-loss temperature.

^d^Weight percent of the residue at 600 °C.

^e^Degree of crystallinity.

^f^Insoluble.

^g^Nematic.

### Extrusion

The powdered TLCP was dried in a vacuum oven at 60 °C for 24 h and spun through a die of a capillary rheometer between 240 and 245 °C. The standard die diameter was 0.50 mm, and the mean residence time in the capillary rheometer was ~ 2–3 min. From a capillary rheometer, the spinning speed of the fiber was 15 m/min. The amount of the polymer for spinning was approximately 20–30 g at a time.

### Characterization

The thermal properties of the samples were investigated by differential scanning calorimetry (DSC), and thermal gravimetric analysis (TGA) was used to measure thermal stability using a DuPont 910 instrument (New Castle, DE, USA). All experiments were conducted under N_2_ atmosphere, and the heating scan rate was 20 °C/min. DSC and TGA were scanned to 30–350 °C and 30–700 °C, respectively.

Wide angle X-ray diffraction (XRD) values were obtained at 25 °C using a Rigaku (D/Max-IIIB) X-ray diffractometer equipped with Ni-filtered Cu-Kα radiation (Tokyo, Japan). The scan speed was 2°/min in the 2θ = 2°–35°. The LC mesophase was investigated using a polarization microscope equipped with a Mettler FP-5 hot stage (Leitz, Ortholux, Lahn-Dill-Kreis, Germany) at various temperature ranges. The fractured section of the spun fiber was observed using a field scanning electron microscope (SEM, JEOL JSM-6500F, Tokyo, Japan). To improve the conductivity, a fractured surface was sputtered with gold using an SPI sputter coater.

The mechanical properties of the fibers were obtained at a crosshead speed of 20 mm/min at room temperature using an Instron mechanical tester (Model 5564) (New York, USA). The experimental errors of the ultimate tensile strength and initial modulus were within ± 1 MPa and ± 0.05 GPa, respectively, the values of the large error range were discarded, and the result was obtained with the average value of at least 10 samples.

## Experimental results

### Thermal behaviors

TLCP fibers were heat-treated at an annealing temperature from 190 to 250 °C to measure thermal properties, liquid crystalline mesophase, and degree of crystallinity (DC) during the same heat treatment time (3 h). These properties were compared for different temperatures. Table 2 summarizes the physical properties according to various annealing conditions. The glass transition temperature (*T*_*g*_) of the as-spun fiber was 82 °C, but a gradual increase in the annealing temperature from 190 to 230 °C for 3 h each gradually increased *T*_*g*_ to 98 °C. *T*_*g*_ is known to depend on the flexibility and stiffness of polymer chains and is closely related to chain interactions and variations in free volume. If the DC is increased by heat treatment in the main chain of a rigid-rod type structure, the segmental motion becomes difficult and the *T*_*g*_ value increases^23^. However, even after heat treatment at 250 °C, *T*_*g*_ did not increase and showed a constant value (97 °C) (see Table 2).

**Figure 2:**
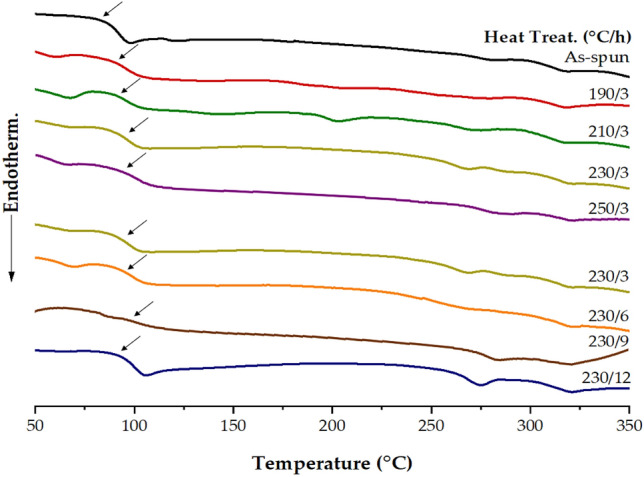
DSC thermograms of TLCPs with various heat treatment conditions.

The melting transition temperature (*T*_*m*_) of the as-spun fiber was observed at 277 °C. The *T*_*m*_ values increased with the annealing temperature, as shown in Table 2. When the annealing temperature was increased to 230 °C, the *T*_*m*_ showed a constant value of approximately 280 °C, but when the annealing temperature was raised to 250 °C, the *T*_*m*_ was 287 °C. The isotropic transition temperature (*T*_*i*_) exhibited a behavior similar to that of *T*_*g*_ and *T*_*m*_. In the case of the as-spun fiber, the *T*_*i*_ was 315 °C, but when heat-treated to 230 °C, it increased to 322 °C, and this value was constant even when heat-treated at 250 °C, as shown in Table 2. Figure 2 shows the DSC thermogram for various heat treatment conditions. The peak intensity of *T*_*i*_ was relatively weaker than that of *T*_*m*_. This is because the amorphous isotropic transition caused by heating is relatively weaker than the transition of *T*_*m*_ in the crystalline state.

The Δ*T*_*i*−*m*_ value representing the stable degree of the LC mesophase showed the largest temperature range of 42 °C when heat treated at 230 °C for 3 h. Increasing this value is useful for spinning because the LC state is more stable over a wide temperature range, and also for a wider range of applications for TLCP fiber use.

The TLCP fibers were very sensitive to the heat treatment conditions and their thermal properties (*T*_*g*_, *T*_*m*_, and *T*_*i*_*)* increased under certain annealing temperature conditions. These results have already been obtained in several studies. Jin et al.^24^ explained the relationship between the thermal properties of TLCP and the structure of monomers according to various heat treatment conditions.

**Figure 3:**
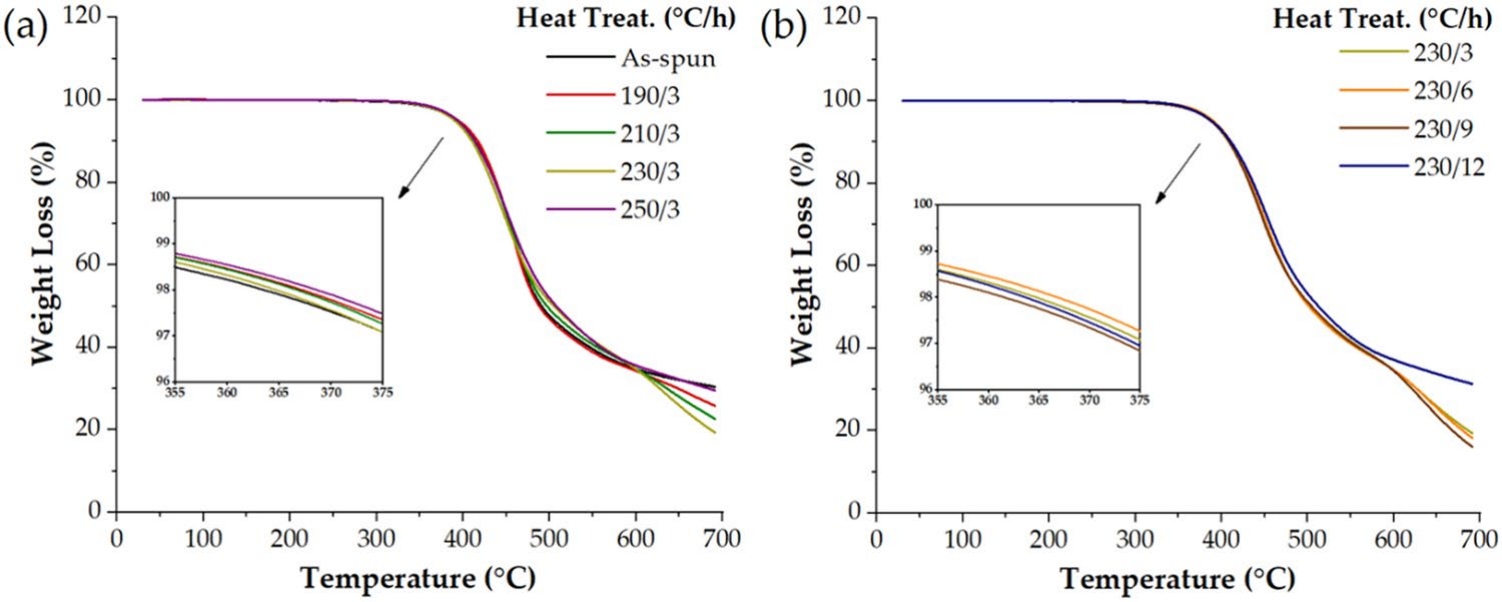
TGA thermograms of TLCPs. (**a**) Various heat treatment temperatures at a constant heat treatment time, (**b**) various heat treatment times at a constant heat treatment temperature.

Table 2 also shows the thermal stability of the TLCP fibers under various annealing conditions. All TLCP fibers showed almost the same thermal stability depending on the annealing temperature. That is, even when heat treatment was performed from 190 to 250 °C for the same period of 3 h, similar results were obtained at the initial decomposition temperature (*T*_*D*_^*i*^) of 363–367 °C. This is due to the acetic acid generated as the polymer reaction proceeds in the molten state during the TGA scanning. The temperature range in which the decomposition products were mainly volatilized by heating was usually around 400 °C, as shown in Fig. 3a. The values of weight residue at 600 °C (*wt*_*R*_^600^) also appeared in the range of 34–36%, regardless of the annealing temperature.

We used TLCP fibers to investigate the thermal properties obtained by heat treatment at 190–250 °C for the period of 3 h. In the results obtained above, the 230/3 sample exhibited several advantages regarding the physical properties of the fiber according to the heat treatment compared to other samples. This sample is easy to process because of its low melting point, and its wide liquid crystalline range (42 °C) enables to verify various physical properties. Therefore, the thermal properties of the 230/3 fibers were further investigated while increasing the annealing time from 3 to 12 h for the same temperature of 230 °C.

Table 2 summarizes the properties of the 230/3 fibers obtained according to changes in annealing time at the same temperature. *T*_*g*_ did not change even when the annealing time was increased from 3 to 6 h, whereas it increased from 98 to 102 °C after heat treatment for 9 h. In general, *T*_*g*_ values increase with increasing annealing time and temperature. In this case, *T*_*g*_ was increased by 20 °C compared to the as-spun fiber. It is well known that the molecular weight increases through the heat treatment process. The high *T*_*g*_ value of the 230/9 sample with increased molecular weight under moderate heat treatment conditions is due to the stiffness of the TLCP main chain and the reduced chain mobility. This is a result of the high rotational barrier to the linkage bonds caused by the diethoxy side groups, which are symmetrically substituted in the terephthaloyl unit, and the presence of major rigid rod-like monomers such as HQ and HBA structures that hinder segment movement^23–25^. However, when the annealing time reached 12 h, *T*_*g*_ suddenly reduced to 93 °C.

The *T*_*m*_ and *T*_*i*_ of the heat-treated fibers exhibited the same values even after heat treatment for up to 6 h. After 9 h of heat treatment, *T*_*m*_ and *T*_*i*_ slightly increased by 5 °C (286 °C) and 3 °C (325 °C), respectively. However, after 12 h of annealing, *T*_*m*_ and *T*_*i*_ decreased by 9 °C (276 °C) and 7 °C (318 °C), respectively. This result is due to the deterioration of thermal properties due to excessive heat treatment. Such excessive heat treatment may interfere with the molecular orientation or crystallization of mesogens^19, 21, 23^. From the above results, the optimal condition for the heat treatment of TLCP fibers was 9 h at 230 °C. The Δ*T*_*i*−*m*_ showed a constant value of 41–42 °C regardless of the annealing time, and *T*_*D*_^*i*^ indicating thermal stability was also constant in the range of 363–366 °C. The values of *wt*_*R*_^600^ also appeared in the range of 35–36%, regardless of the annealing conditions (Table 2). The reason for the similarity of the thermal stabilities is that they decompose first at a low temperature due to the low thermal stability of the dialkoxy side groups, regardless of the heat treatment conditions. Figure 3b shows the TGA thermograms for various annealing times at 230 °C.

In conclusion, when heat treatment was performed at various times and temperatures, the thermal properties were improved compared to those of as-spun fibers. However, the thermal stability was almost constant regardless of the annealing conditions.

### LC Mesophase

**Figure 4:**
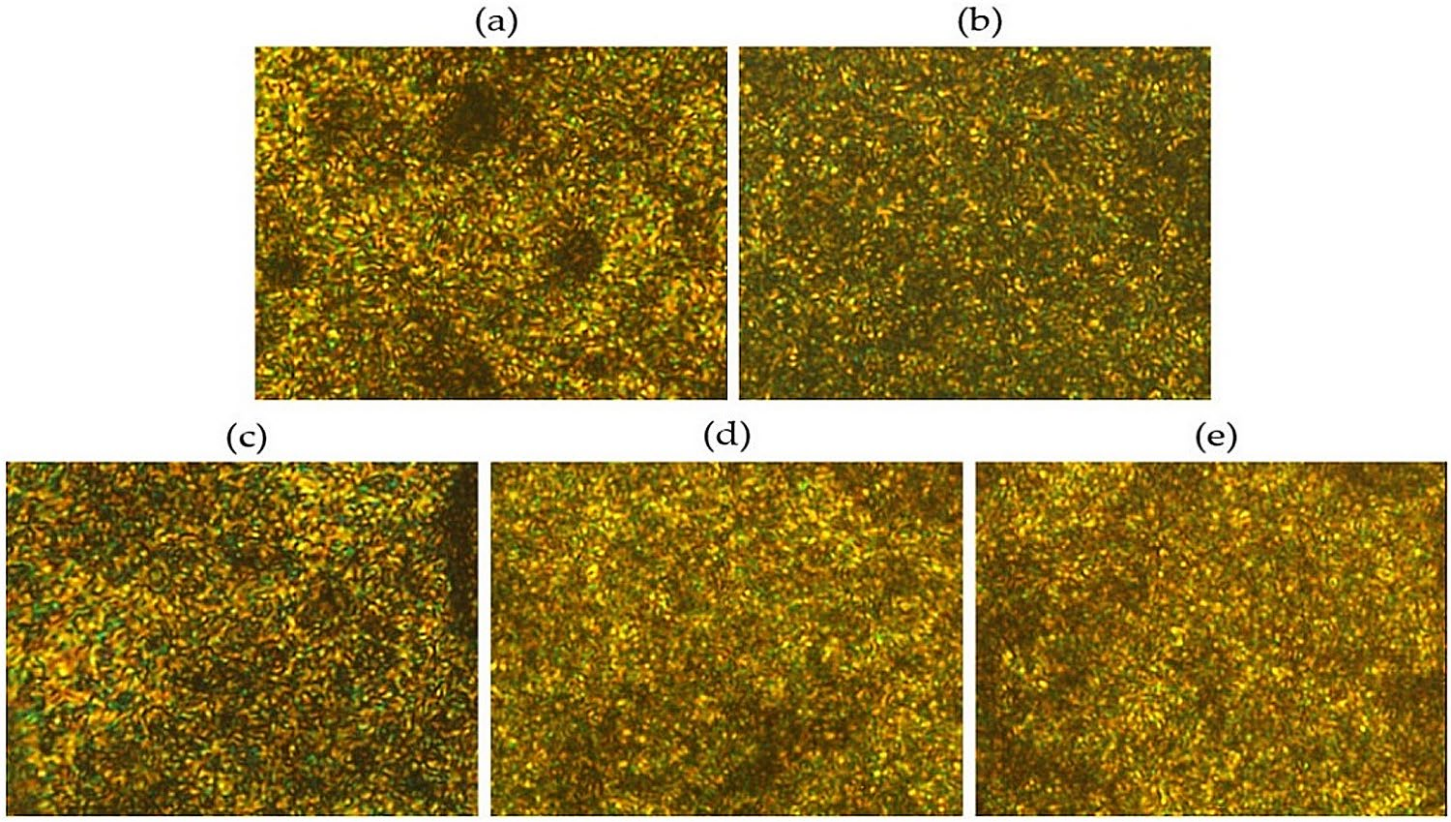
Polarized optical micrographs of TLCP fibers with various heat treatment temperatures (magnification 200×): (**a**) as-spun, (**b**) 190/3, (**c**) 210/3, (**d**) 230/3, and (**e**) 250/3.

The LC mesophase can be observed between *T*_*m*_ and *T*_*i*_ using a polarized microscope while changing the temperature^26, 27^. Figure 4 shows the LC phase observed during heat treatment for the period of 3 h at various temperatures. To obtain better micrographs, a heating and cooling process was performed in the temperature range where the LC phase appeared, and all the LC mesophase photos presented were obtained by a heating process.

**Figure 5:**
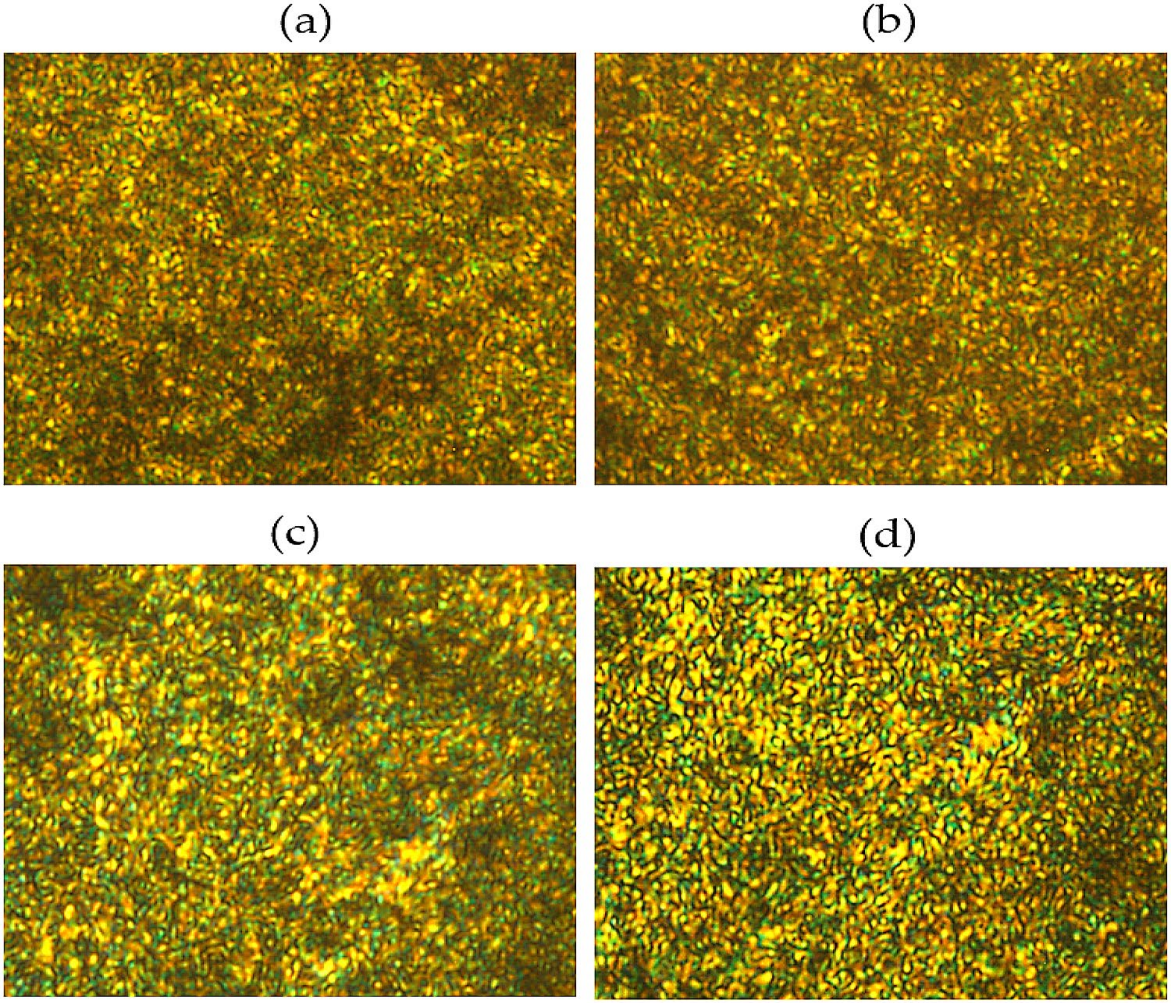
Polarized optical micrographs of TLCP fibers with various heat treatment times (magnification 200×): (**a**) 230/3, (**b**) 230/6, (**c**) 230/9, and (**d**) 230/12.

All LC mesophases of TLCP heat-treated at various periods for the same temperature (230 °C) exhibited a thread-like nematic texture, as shown in Fig. 5^28^. The thread textures were not clearly visible on the nematic phase we obtained, which was mainly due to the high molecular weight or poor flow of the mesogens above *T*_*m*_. The stability of the LC mesophase depends on the stiffness and aspect ratio of the mesogen units. If the mesogen in the main chain of the TLCP is a straight and rigid-rod type, the mesophase of the LCP can be stabilized. However, when heating a structure that contains an excessive amount of HBA, such as the structure used in this study, the flow of molecules deteriorates, hindering the obtainment of clear LC textures^29, 30^.

### Degree of crystallinity by XRD

The crystallinity of TLCP on the annealing effect has been extensively studied. In particular, Blackwell et al.^31^ reported the effect of substituents in the main-chain TLCP according to the heat treatment conditions. They announced that the side order of the random copolyester was improved and the random sequence was crystallized into ordered crystals with a higher melting temperature through the annealing process. By comparing the intensities of the XRD diffraction patterns and the peak intensities before and after annealing, it is possible to investigate the change in crystal size and crystal structure.

In general, the results of increasing the molecular weight and DC through the heat treatment process have already been reported^31^. The DC was determined from the ratio of the peak areas of the crystalline and amorphous regions. The DC values were measured and are summarized in Table 2. The DC of the as-spun fiber was 27%, but when the heat treatment temperature was increased from 190 to 250 °C for 3 h, the DC gradually increased from 30 to 37%. In addition, these DCs exhibited an almost constant value of 36–37% even when the heat treatment time was increased from 3 to 9 h for the same temperature of 230 °C. However, this value decreased to 33% after 12 h of heat treatment. This result can be explained by the reduction due to excessive heat treatment.

**Figure 6:**
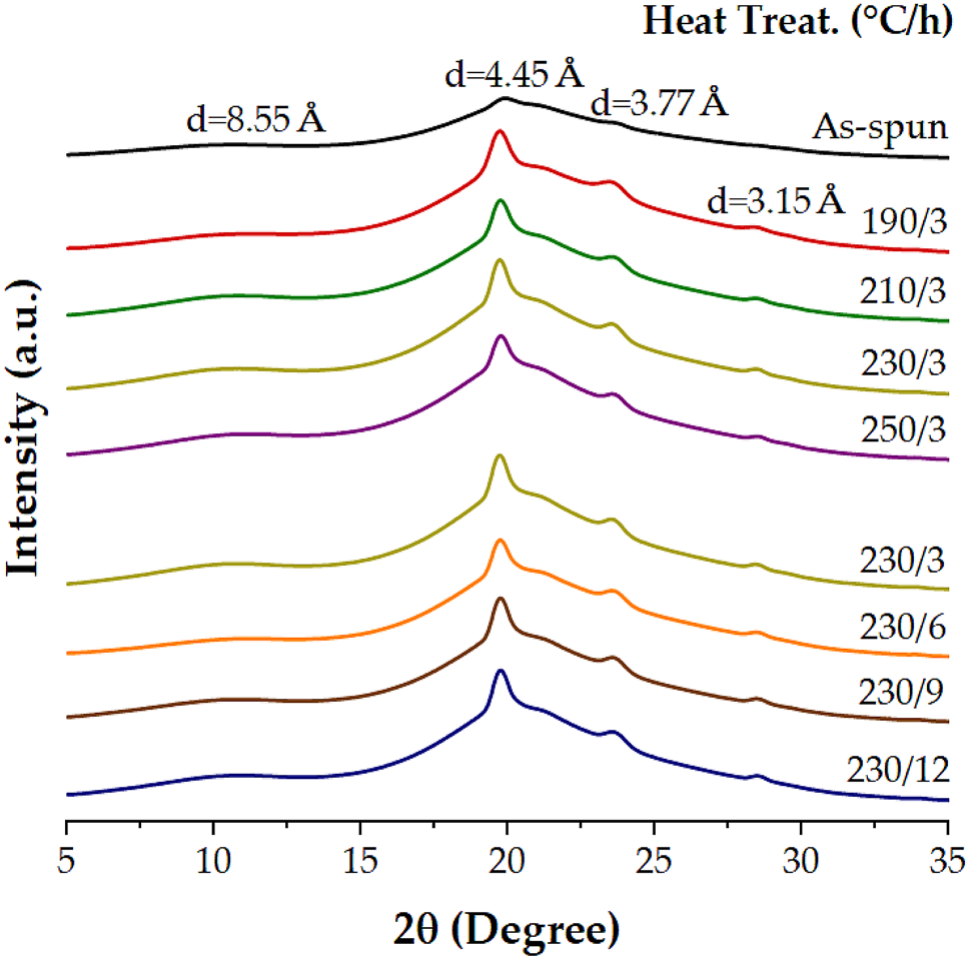
XRD patterns of TLCP fibers with various heat treatment conditions.

Figure 6 shows the effects of annealing on the XRD profile. In the as-spun XRD results, a very weak and broad peak was observed at *d* = 8.55 Å (2θ = 10.34°), and a mid-intensity peak was observed at *d* = 4.45 Å (2θ = 19.92°). In addition, a very small peak was observed at *d* = 3.77 Å (2θ = 23.58°). On the contrary, as the heat treatment temperature and time increased, the intensity of the peak gradually increased and a new peak was also observed. It was observed that the three peaks in the as-spun spectrum were in the same position for sample 190/3, with an increase in the intensity of these peaks. A small new peak was observed at d = 3.15 Å (2θ = 28.18°). This peak is considered to be a peak generated in the process of crystallization by heat treatment. For these peaks, when the annealing temperature was increased to 250 °C for the same period of 3 h, the intensity of the peak also increased. In addition, even if the annealing time was increased from 3 to 9 h at 230 °C, the XRD peak was almost the same. However, when the annealing was increased to 12 h, the intensity of the peak slightly decreased. The results observed in Fig. 6 are consistent with those in Table 2.

### Fiber morphology

Conventional fibers produced from ordinary semi-crystalline polymers are complex aggregates of strong, highly ordered microfibrils and macrofibrils that are usually separated by a weak boundary. There are several methods to produce fibers, but the final product must always exhibit a well-developed fibrous morphology to provide excellent thermal and mechanical properties^32, 33^.

**Figure 7:**
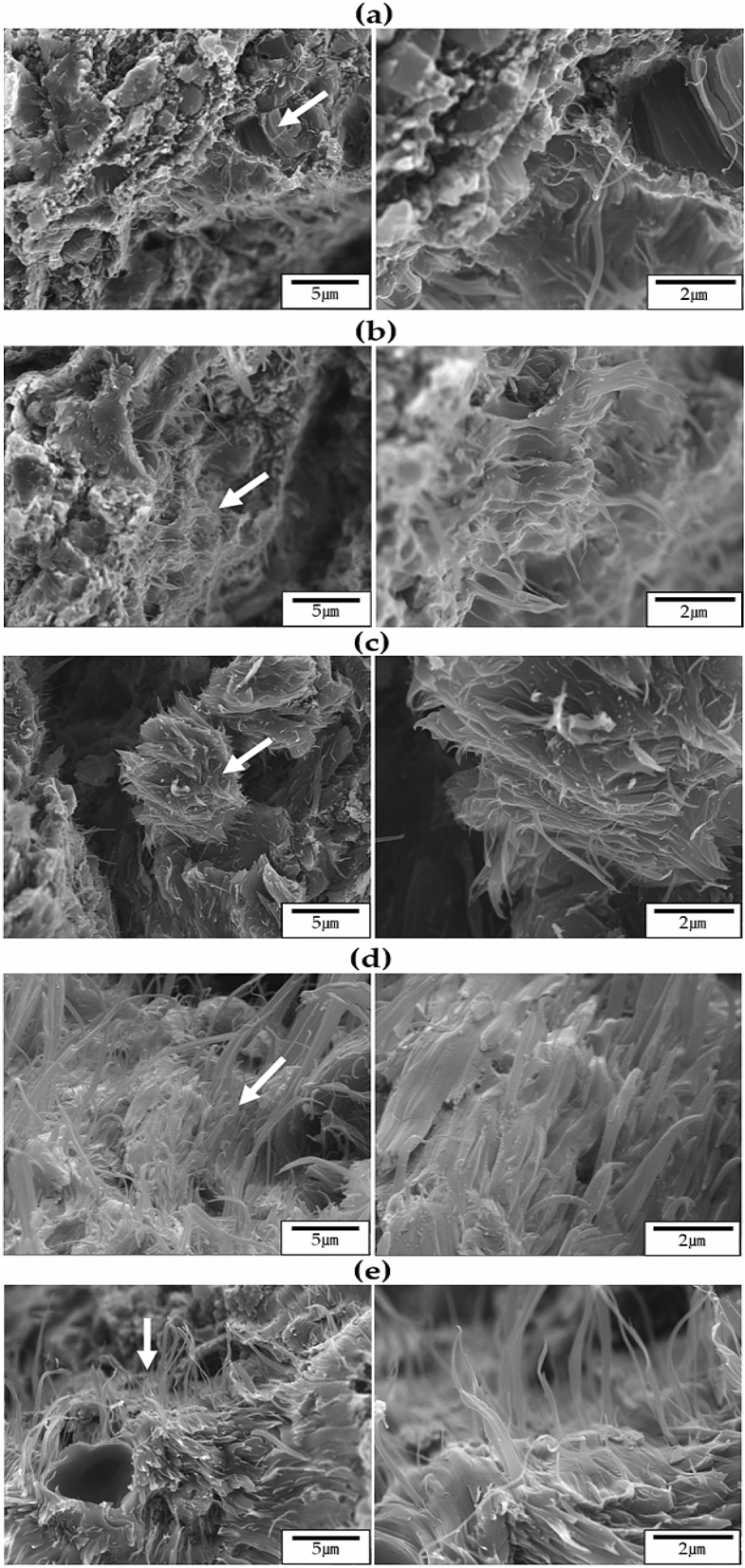
SEM micrographs of TLCP fibers with various heat treatment temperatures: (**a**) as-spun, (**b**) 190/3, (**c**) 210/3, (**d**) 230/3, and (**e**) 250/3. Micrographs with increasing magnification levels fr om left to right.

The TLCP fiber was vertically broken in liquid nitrogen, and the cross section was examined by SEM. Figure 7 shows SEM micrographs of the fibers heat-treated under various annealing conditions. In the case of as-spun fibers, fibrous morphology was observed in some parts, as shown in Fig. 7a, but small spherical particles that were not well developed were observed in most areas, and these particles were evenly distributed. When the as-spun fibers were heat-treated at 190 and 210 °C for the same period of 3 h, fibrous phases were observed in a wider area than the as-spun fibers. However, the observed fibrous morphology was not developed, and there was no significant difference from the as-spun fiber in its overall shape (see Fig. 7b,c). However, as the annealing temperature increased to 230 °C, several extruded fiber morphologies were observed, as shown in Fig. 7d. In addition, very well-developed fine fibrous morphologies were observed in a large area. This result was similarly observed for the fiber heat-treated in the 250/3 sample (Fig. 7e). From these results, the fibers heat-treated at relatively high temperatures were observed to have a well-developed highly oriented fibrous morphology, whereas fibers annealed at low temperatures showed poor orientation and no fibrous morphology. It was also found that the fibers obtained under the optimal heat treatment conditions exhibited excellent fibrous morphology.

**Figure 8:**
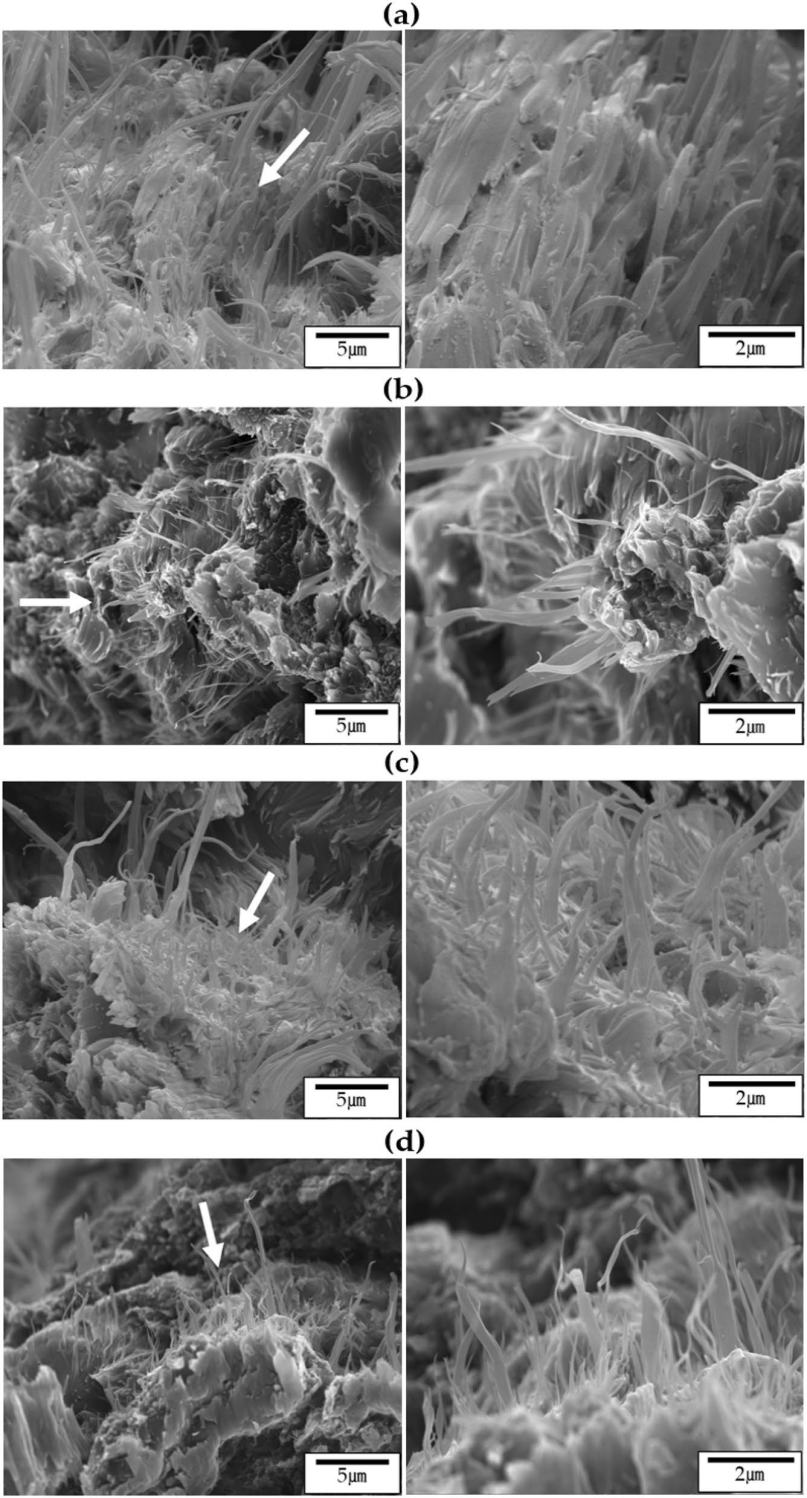
SEM micrographs of TLCP fibers with various heat treatment times: (**a**) 230/3, (**b**) 230/6, (**c**) 230/9, and (**d**) 230/12. Micrographs with increasing magnification levels from left to right.

The morphologies of the fibers with increasing annealing time at a constant temperature of 230 °C were also observed, and the results are shown in Fig. 8. When the heat treatment was increased from 3 to 9 h, micro-sized fibers were observed over a large area, and the distribution of these fibers was constant regardless of the heat treatment time (Fig. 8a–c). This tendency did not change even when the heat treatment time was 12 h, as shown in Fig. 8d. The thickness of the fibers obtained through various annealing processes was mostly less than the micron size, and as the heat treatment time increased, the ''skin–core'' morphology is often observed when spinning TLCP fibers. This is because TLCPs containing a straight and rigid rod-like molecular structure have different molecular orientations when processed by high heat and pressure in the 'skin' and 'core' regions. In the skin area, the molecular domains are parallel to the flow direction, but in the core area, where heat and pressure do not reach the core area, the molecular orientation cannot maintain a constant direction^34–36^. These results were reported for TLCP, and also for polyester and nylon 6^37^.

**Figure 9:**
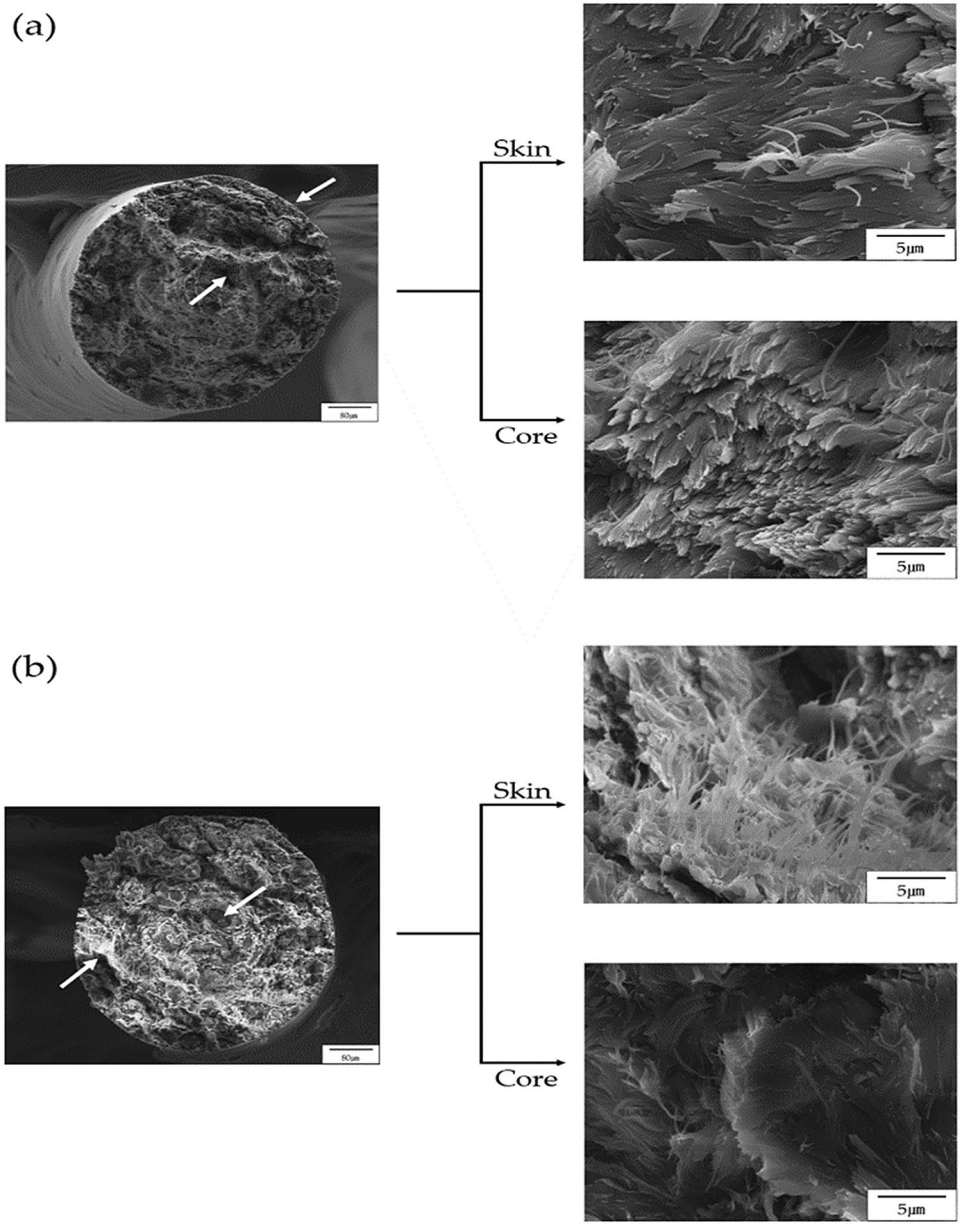
SEM micrographs of the skin–core morphology in (**a**) as-spun and (**b**) 230/9 fibers.

SEM micrographs of as-spun and 230/9 samples are shown in Fig. 9 to observe the skin and core morphology under different heat treatment conditions. In the case of as-spun fibers, as shown in Fig. 9a, some fibers were observed in a very small part of the skin area, but most of them did not exhibit a fibrous shape. In the core region, almost all of them were kept in a clustered shape, and a slightly pulled structure was observed, and this morphology was not significantly different from the results observed in the skin. Significant amounts of LCP were observed in the form of aggregates of elliptical and spherical particles. These results were similar to those already obtained in Fig. 7a.

Figure 9b shows an enlarged SEM image of the cross section of the 230/9 sample. A particle shape was observed in small areas of the skin, but a microfiber phase with a large aspect ratio was observed in most areas. The diameters of these fibers range from tens to hundreds of nanometers. Figure 9b also shows the core region of the same sample. A small amount of fibrous phase was also observed in the core area, but compared to the fibers obtained from the skin area, these fibers were not well defined and the fibrous morphology was thicker and shorter. This skin–core morphology seems to be due to the difference in shear and expansion flow ranges occurring during the LCP fiber processing step.

**Figure 10:**
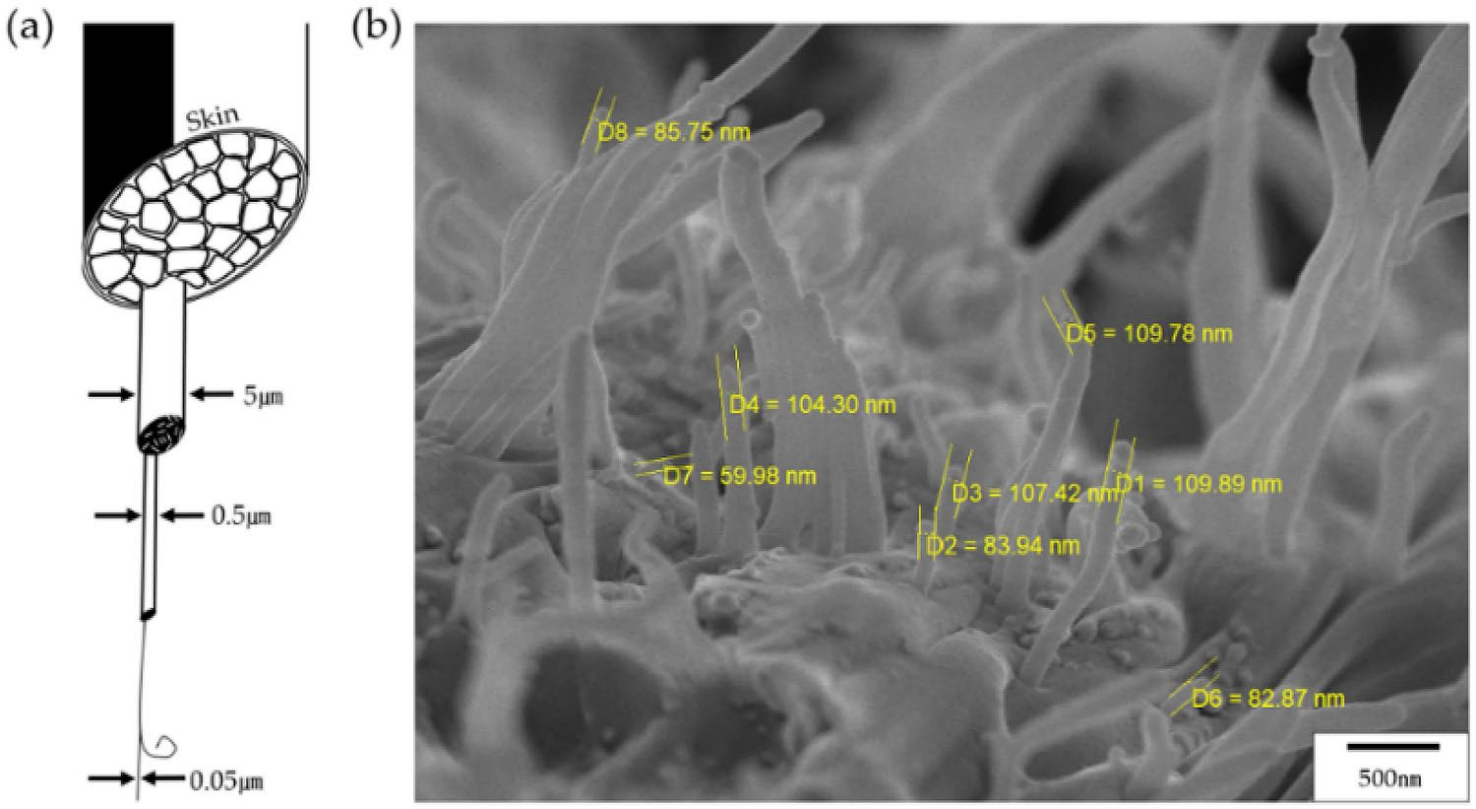
(**a**) Hierarchical structures in TLCP fibers^38^ and (**b**) SEM micrograph of 230/9 fiber.

Because of the rigid rod-like mesogenic structure, LCP fibers exhibit a very well-developed microfiber morphology. In 1986, Sawyer and Jaffe^38^ presented a hierarchical model of the morphology of already spun TLCP fibers. The structural hierarchical model observed for all oriented TLCP structures is shown in Fig. 10a. In the case of macrofibers, the diameter is approximately 5 μm, and it is approximately 0.5 μm for fibers commonly observed, and the microfibers have a diameter of approximately 0.05 μm (50 nm); thus, these microfibers can be detected on the actual peeled fiber and also on the fracture surface. These results have already been identified in Figs. 7, 8 and 9. For a more detailed observation, we examined the diameter of each fiber using a fiber of 230/9. As can be seen in Fig. 10b, the diameter of the 230/9 fiber was in the range of approximately 60–110 nm (0.06–0.11 μm), and this value was also observed in the fiber under different heat treatment conditions.

Numerous well-developed microfibers are observed in the skin area because the orientation of the TLCP fibers is considerably influenced by the draw flow occurring in the die of the spinneret and the shear flow generated in the spinning process. Spinning is a successful method to produce microfibers with high aspect ratios and highlights the importance of the heat treatment to obtain well-developed microfibers through the morphology of the fibers.

### Mechanical tensile properties

TLCP molecules with a rigid rod-like mesogen form an anisotropic LC phase in the molten state, thus they are easily oriented by external forces. In addition, excellent tensile mechanical properties can be obtained through a heat treatment process known as solid-state polymerization or increasing alignment in an LC state. Therefore, changes in the molecular arrangement or geometric morphology of TLCP fibers by heat treatment have a significant effect on the mechanical properties such as the strength and modulus of the fibers^19, 21, 39^. In Fig. 6 and Table 2, we observe the change in DC according to molecular orientation by heat treatment using XRD.

**Figure 11:**
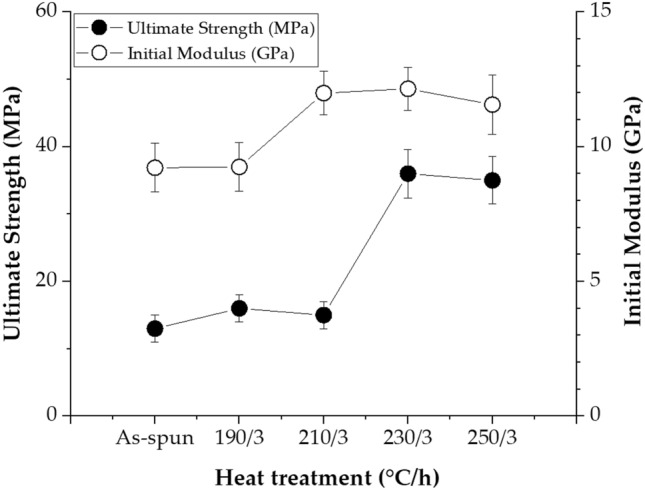
Tensile properties of TLCP fibers with various heat treatment temperatures at a constant annealing time.

Table 3 summarizes the changes in tensile mechanical properties according to various heat treatment conditions. For as-spun fibers, the ultimate tensile strength was 13 MPa, but this value was almost constant (15–16 MPa) even when the annealing temperature was increased to 210 °C for the same period of 3 h. The reason why the tensile strength is particularly low compared to other general TLCP fibers is that the dialkoxy side groups contained in the main chain interfere with the orientation of the molecules and the intermolecular bonding between the side groups itself is poor. However, these results were considerably improved by the heat treatment process. When the annealing temperature was increased to 230 °C, a value of 36 MPa was observed, but this value decreased slightly at 250 °C to 34 MPa. In the case of the initial modulus, the results were similar to those of the ultimate tensile strength. The initial modulus of the as-spun fiber was 9.22 GPa, but when the annealing temperature was increased from 190 to 230 °C for the same period of 3 h, the initial modulus increased from 9.25 to 12.15 GPa. However, this value was 11.56 GPa, which slightly decreased at the annealing temperature of 250 °C. Figure 11 shows the variation in mechanical properties according to various annealing temperatures for the same period of 3 h.

**Table 3:** Tensile properties of TLCP fibers.

Heat treat. (°C/h)	Ult. str. (MPa)	Ini. mod (GPa)	E.B. (%)
As-spun	13	9.22	10
190/3	16	9.25	12
210/3	15	11.99	10
230/3	36	12.15	10
250/3	34	11.56	10
230/3	36	12.15	10
230/6	72	12.77	10
230/9	102	16.11	12
230/12	58	14.66	10

**Figure 12:**
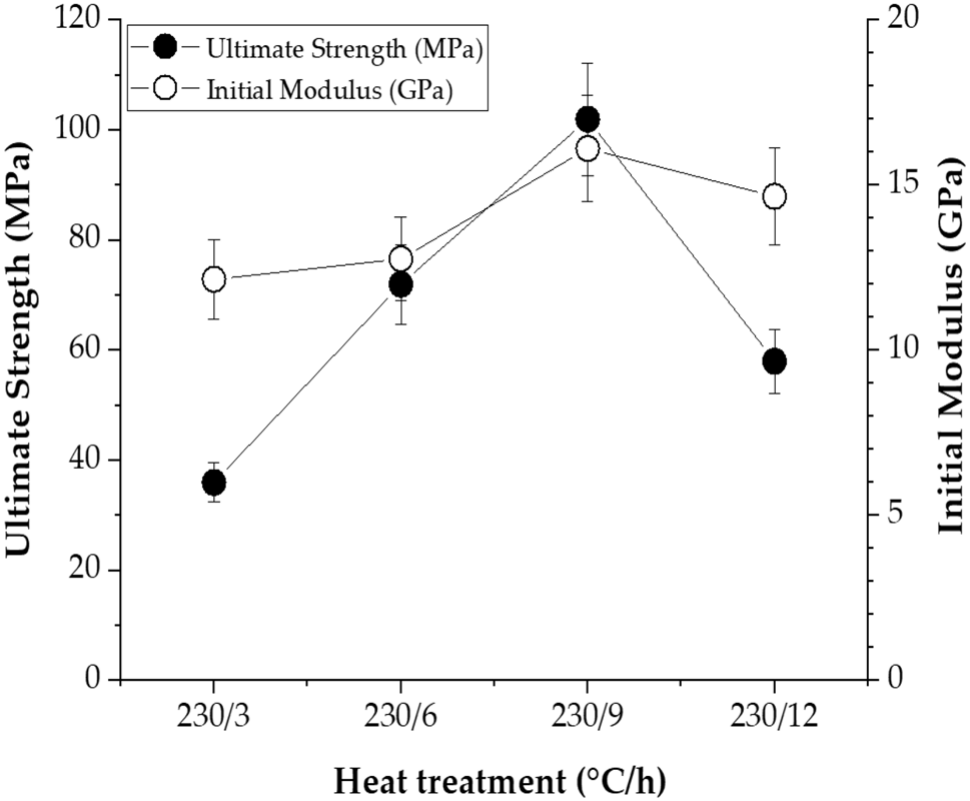
Tensile properties of TLCP fibers with various heat treatment times at a constant annealing temperature.

In a previous study, the 230/3 fiber provided the best mechanical properties, thus we used this sample to observe the results while varying the annealing time at the same annealing temperature to obtain an optimal value. When the heat treatment time was increased from 3 to 9 h at 230 °C, the tensile strength and initial modulus increased to 102 MPa and 16.11 GPa, respectively, as shown in Table 3. These values increased by 7.8 and 1.7 times, respectively, compared to as-spun fibers. However, when the annealing time reached 12 h, the mechanical properties were reduced, and the ultimate tensile strength and initial modulus were 58 MPa and 14.66 GPa, respectively. Elongation break (EB) was mostly 10–12% regardless of the heat treatment conditions. These low EB values are mainly observed in polymers containing straight and rigid rod-like mesogens. Figure 12 shows the mechanical properties according to various annealing times for the same temperature of 230 °C.

In conclusion, in terms of mechanical tensile properties, optimal results were obtained for 230/9 fiber samples. As described above, this increase in mechanical properties is due to an increase in molecular weight and orientation of molecular chains by heat treatment. These results were confirmed by the XRD and SEM results.

## Conclusions

TLCP was synthesized in a molar ratio of ETA:HQ:HBA = 1:1:3 using a melt polymerization method. The synthesized TLCP showed a nematic texture in the shape of a thread and was spun from the capillary rheometer above the melting temperature. The obtained fibers were heat treated for various temperatures and times, and the thermo-mechanical properties, liquid crystalline mesophase, and morphology were investigated according to the heat treatment conditions.

In general, the thermo-mechanical properties increased with increasing heat treatment temperature and time, and the LC properties were maintained in all samples regardless of the heat treatment conditions. The skin–core morphology generated during the spinning process was also observed. The diameter of the spun fibers ranged from approximately 60–110 nm, and this value was also observed for fibers obtained under different heat treatment conditions.

From the results of this study, the TLCP fiber obtained in sample 230/9 showed the best results. In particular, in terms of mechanical properties, the 230/9 sample increased the ultimate tensile strength by 7.8 times and the initial modulus by 1.7 times, compared to the as-spun fibers.

In this study, we designed the TLCP structure such that it could be synthesized and spun at low temperature. We also proposed a method for maximizing the thermo-mechanical properties while maintaining the liquid crystallinity of the obtained fiber through an appropriate heat treatment process.
